# Current Advance of Therapeutic Agents in Clinical Trials Potentially Targeting Tumor Plasticity

**DOI:** 10.3389/fonc.2019.00887

**Published:** 2019-09-10

**Authors:** Xiao-Guang Yang, Lan-Cao Zhu, Yan-Jun Wang, Yan-Yu Li, Dun Wang

**Affiliations:** Key Laboratory of Structure-Based Drug Design & Discovery of Ministry of Education, Shenyang Pharmaceutical University, Shenyang, China

**Keywords:** tumor plasticity, cancer stem cells, vasculogenic mimicry, extracellular matrix, tumor microenvironment, targeting

## Abstract

Tumor plasticity refers to tumor cell's inherent property of transforming one type of cell to different types of cells. Tumor plasticity is the main cause of tumor relapse, metastasis and drug resistance. Cancer stem cell (CSC) model embodies the trait of tumor plasticity. During carcinoma progression, epithelial-mesenchymal transition (EMT) plays crucial role in the formation of CSCs and vasculogenic mimicry (VM) based on epithelial-mesenchymal plasticity. And the unique tumor microenvironment (TME) not only provides suitable niche for CSCs but promotes the building of CSCs and VM that nourishes tumor tissue together with neoplasm metabolism by affecting tumor plasticity. Therapeutic strategies targeting tumor plasticity are promising ways to treat malignant tumor. In this article, we discuss the recent developments of potential drug targets related to CSCs, EMT, TME, VM, and metabolic pathways and summarize drugs that target these areas in clinical trials.

## Introduction

The universal methods for cancer treatment include surgery, radiotherapy, and chemotherapy. Chemotherapy is the principle modality for the treatment of malignant tumor, especially tumors in the late stages. Despite significant improvement of cancer chemotherapy in clinical practice, there are still many obstacles that chemotherapeutic drugs must overcome: (1) lack of effective treatments for metastatic tumors; (2) ineffectiveness in killing drug-resistant tumor cells; and (3) lack of new targets based on the characteristics of neoplasm, such as tumor plasticity.

Tumor plasticity prompts tumor cells to differentiate into a variety of cell types to adapt to different environment (1). Emerging evidence suggested that tumor plasticity played critical roles in the emergence of drug resistance and the promotion of tumor growth, invasion and metastasis. Therefore, there is an urgent need to develop new therapeutic agents to target tumor plasticity.

**Figure 1:**
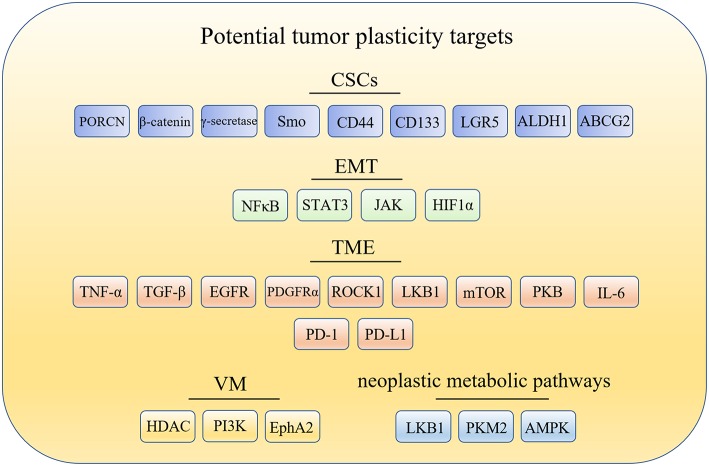
Potential drug targets related to tumor plasticity. CSCs, cancer stem cells; EMT, epithelial-mesenchymal transition; TME, tumor microenvironment; TME, tumor microenvironment; PORCN, porcupine; Smo, smoothened; LGR5, leucine-rich repeat containing G protein-coupled receptor 5; ALDH1, aldehyde dehydrogenase1; ABCG2, breast cancer-resistant protein; NF-κB, nuclear factor-kappa B; JAK/STAT3, the Janus kinase/signal transducer and activator of tran-ions 3; HIF1α, hypoxia-inducible factor 1α; TNF-α, tumor necrosis factor alpha; TGF-β, transforming growth factor-β; EGFR, epidermal growth factor receptor; PDGFRα, platelet derived growth factor receptor alpha; ROCK1, Rho kinase1; LKB1, liver kinase B1; mTOR, mammalian target of rapamycin; PKB/Akt, protein kinase B; IL-6, interleukin-6; PD-1, programmed cell death receptor-1; PD-L1, programmed cell death-ligand 1; HDAC, histone deacetylases inhibitor; PI3K, phosphatidylinositide 3-kinases; MMPs, matrix metalloproteinases; Eck/EphA2, epithelial cell kinase; LKB1, liver kinase B1; PKM2, pyruvate kinase M2; AMPK, AMP-activated protein kinase.

The cancer stem cells (CSCs) model offers one explanation for tumor plasticity. The CSCs model revealed that only a minority of tumorigenic cells contribute to tumor growth and progression. However, there are many other aspects closely related to tumor plasticity. For example: (1) epithelial-mesenchymal transition (EMT), which contributes to the phenotypic plasticity and promotes cancer metastasis; (2) tumor microenvironment (TME), which contains extracellular matrix (ECM) and cells such as fibroblasts, endothelial and immune cells that are the primary source of signals to and from the tumors; (3) vasculogenic mimicry (VM), which is a microcirculation that is independent of angiogenesis in aggressive primary and metastatic tumors and comprised of non-endothelial cell generated by tumor cells and ECM; and (4) neoplastic metabolic pathways, that mainly include glycolysis and oxidative phosphorylation (OXPHOS). Changes of metabolic pathways between glycolysis and OXPHOS in cancer cells is prevalent during tumorigenesis and metastasis. Hence, targeting glycolysis and OXPHOS is essential to wipe out metabolic plasticity in cancer cells. Here, the potential targets related to tumor plasticity was summarized in Figure 1. In this mini review, we summarize the recent advances in anticancer compounds targeting CSCs, ETM, TEM, VM formation, and metabolic pathways, which is associated with tumor plasticity.

## Therapeutic Targeting of CSCs

The concept of CSCs was proposed several decades ago. The existence of CSCs has been confirmed by lineage tracing and cell ablation experiments in tumors (2–6). Similar to normal stem cells, a small subset of CSCs could proliferate and differentiate into a wide variety of cell types to sustain and promote tumor growth. The characteristic of tumor plasticity in CSCs is that CSCs could differentiate in different directions. The CSCs model provides a new explanation for the metastasis and recurrence of malignant tumors. CSCs have also been recognized as a major driver of tumor growth, metastasis and chemotherapeutic resistance. Therefore, CSCs has become crucial targets for cancer treatment. The ways to eliminate CSCs mainly consist of two aspects (7): (1) inhibition of key CSCs signaling pathways, including Wnt pathway, porcupine (PORCN) pathway and Hedgehog (Hh) pathway (8, 9); and (2) ablate CSCs by targeting CSC surface markers, such as CD133, CD44, (leucine-rich repeat containing G protein-coupled receptor 5) LGR5, (aldehyde dehydrogenase1) ALDH1, and breast cancer-resistant protein (BCRP; ABCG2). Table 1 summarizes drugs that target CSCs in recent clinical trials.

**Table 1:** Potential drugs targeting CSCs in clinical trials.

**Drug**	**Mechanism of action**	**Condition or disease**	**Phase**	**References**
WNT-974	PORCN inhibitors	Colorectal cancer and melanoma	I	(10)
ETC-159	PORCN inhibitors	Advanced solid tumors	I	(11, 12)
CGX-1321	PORCN inhibitors	Refractory solid tumors and advanced gastrointestinal cancers	I	(13)
RXC-004	PORCN inhibitors	Solid tumors	I/II	(14)
BC-2059	β-catenin inhibitors	Desmoid tumors	I	(15)
E-7386	CREB-binding protein (CBP)/β-catenin interaction inhibitors	Solid tumors	I	(16)
AL-101	γ-secretase inhibitors	Adenoid cystic carcinoma	II	(17)
Vismodegib	p-glycoprotein inhibitors Breast cancer-resistant protein inhibitors Smo receptor antagonists	Basal cell carcinoma, other cancers	Launched in 2012	(18)
Sonidegib phosphate	Smo receptor antagonists	Basal cell carcinoma, other cancers	Launched in 2015	(19)
Patidegib	Smo receptor antagonists	Sarcoma, basal cell carcinoma	III	(20, 21)
Taladegib	Smo receptor antagonists	Adenocarcinoma, solid tumors	I/II	(22, 23)

Therapeutic agents targeting Wnt signaling pathway in clinical trials include porcupine (PORCN) inhibitors, β-catenin inhibitors and antibodies against Wnt signaling molecules (24). Among these, PORCN inhibitors gradually became research focus of antitumor drugs. **WNT-974**, an orally first-in-class PORCN inhibitor, is a pyridinyl acetamide derivative that target Wnt signaling to inhibit the expression of Wnt related genes and Wnt-dependent LRP6 phosphorylation. **WNT-974** showed significant growth inhibitory effect on Wnt-driven neoplasms, such as pancreatic cancer and head and neck squamous cell carcinoma. The pharmacokinetics (PK) and pharmacodynamics (PD) of **WNT974** were tested in patients with advanced cancers in phase I clinical trial, and the results showed rapid absorption (median *T*_max_ 1–3 h) and appropriate elimination half-life of 5–8 h. These clinical data demonstrated that **WNT-974** possesses favorable safety profile and potential antineoplastic activity in selected populations (25). Currently, **WNT-974** is being tested in a phase I study for the treatment of solid tumors including colorectal cancer and melanoma (10). In addition, PORCN inhibitor **ETC-159** is in phase I clinical trial for advanced solid tumors, **CGX-1321** is in phase I clinical trials for advanced gastrointestinal cancers and **RXC-004** is in phase I/II clinical trials for the treatment of solid tumors (11–14). Through the inhibition of β-catenin, **Tegavivint** (BC-2059), an anthraquinone derivative and **E-7386** are both being evaluated in phase I clinical trials to treat symptomatic or progressive unresectable desmoid tumors and solid tumors (15, 16).

The small-molecule inhibitors and macromolecule monoclonal antibodies (mAbs) including γ-secretase inhibitors and mAbs to NOTCH receptors have been tested in clinical trials. A small-molecule inhibitor of γ-secretase, which is a key enzyme in NOTCH signaling pathway, **AL-101** with favorable *in vitro* potency and oxidative metabolic stability, is in phase II clinical development for the treatment of adenoid cystic carcinoma bearing NOTCH activated mutations (17). On the other hand, among the therapeutic molecules targeting Hh pathway, smoothened (Smo) receptor antagonists are the most promising molecules (26). A novel small-molecule inhibitor or antagonist of Smo, **Sonidegib phosphate** was launched in 2015 for the treatment of advanced basal cell carcinoma (BCC). **Sonidegib phosphate** exhibited excellent therapeutic effect (roughly 35–60% response rates of patients) in patients with locally advanced, unresectable and metastatic BCC, with high disease control rates and clinical benefit (19, 27). Recent advances in the development of Hh signaling inhibitors include **Vismodegib** (18), which is launched in 2012 for the treatment of patients with advanced BCC; **Patidegib**, which is in phase III clinical trial for reducing the incidence of BCC (20, 21) and **Taladegib**, which is in phase I/II clinical trial) for the treatment of patients with recurrent, advanced solid tumors (22, 23).

Because of the highly plasticity of CSCs in tumors, the identification and eradication of CSCs are difficult. Generally, their identification depends on cell surface markers. CD34, CD44, and CD133 are common examples of CSC-specific surface markers (28). CSC surface markers can mediate adhesion of the cells. A cell surface membrane protein CD133, which was first discovered in hematopoietic stem and progenitor cells, is considered to be one of the common surface markers in multiple stem cells (29). Others like ALDH1 and ABCG2 also play significant roles in the regulation of CSCs (30, 31). Because CSCs drive cancer development, a number of agents targeting the biomarkers of CSCs have been developed (Table 2).

**Table 2:** Potential drugs targeting CSC surface marker in clinical trials.

**Drug**	**Mechanism of action**	**Condition or disease**	**Phase**	**References**
P5	Anti-CD49e/CD29 (integrin α5β1)	Non-small cell lung cancer (NSCLC)	III	(32)
ALM-201	Microtubule inhibitors (binds CD44)	Advanced ovarian cancer and other solid tumors	I	(33)
RO-5429083	Anti-CD44	Acute myeloid leukemia	I	(34)
RG-7356	Anti-CD44	Acute myeloid leukemia	I	(35)
AMC-303	CD44 Antigen Exon 6 (CD44v6) inhibitors	Advanced or metastatic malignant solid tumors of epithelial origin	I/II	(36)
CX-2009	Tubulin polymerization inhibitors Anti-ALCAM (CD166)	Solid tumors	I/II	(37)
Chrysin	ABCG2 inhibitors	Chronic lymphocytic leukemia (CLL)	II	(38)

A novel mAb **P5**, which targets CD49e/CD29, is currently being tested in phase III clinical trials to evaluate its anti-tumor effect, but there are only a few reports about its progress of new clinical trials (32). As a FK506 binding protein like (FKBPL) peptide derivative, **ALM-201** can bind to CD44 and inhibit cancer related pathways, such as DLL4/NOTCH signal pathway as well as inhibit cell migration, tubule formation and angiogenesis. **ALM-201** showed an excellent safety profile and acceptable PK in patients with advanced solid tumors in a phase I dose-escalation study (39). This candidate is currently in phase I clinical trials for the treatment of patients with advanced ovarian cancer and other solid tumors (33). **RO-5429083** and **RG-7356** are both humanized monoclonal antibodies against extracellular domain of CD44 which had been used in phase I clinical studies for the treatment of acute myeloid leukemia and solid tumors (34, 35). In addition, **AMC-303**, a high specific inhibitor of CD44v6, was evaluated as monotherapy to treat patients with advanced epithelial tumors. **AMC-303** was proved to be well-tolerated with a favorable PK profile (*t*_1/2_ of 4–7 h, CL of 40–60 mL/h/kg) (40). At present, **AMC-303** is in phase I/II clinical trials to treat patients with advanced or metastatic malignant solid tumors of epithelial origin (36). A probody drug conjugate **CX-2009** against CD166 is in phase I/II clinical development for the treatment of adult patients with metastatic or locally advanced unresectable solid tumors (37). Furthermore, a recent research reported that **chrysin**, which is an ABCG2 inhibitor, could enhance sorafenib mediated inhibition of cell viability by sustained phosphorylation of ERK1/2 (41). And **chrysin** is being used in phase II clinical trials to treat CLL (38).

## Therapeutic Targeting of EMT

The conversion of cells from epithelial phenotype into mesenchymal phenotype is a critical transformation for embryonic development and during cancer progression. Through EMT process, tumor cells can acquire the ability to disarm anti-tumor defenses in the body, resist apoptosis and antineoplastic drugs, spread through the body and expand the population of tumor cells (42). At the same time, EMT may play an important role in generating CSCs (43). Hence, EMT is an important target for inhibiting tumor metastasis and reducing drug resistance. Various approaches can be used to target the EMT process: (1) targeting the inducing signals in EMT process; (2) reversing EMT to reduce tumor cell aggressiveness; and (3) killing the cells in EMT-like state (44). As one of the key factors of tumor invasion, metastasis and drug resistance, EMT is a promising target for oncotherapy. The following summarized the progress of potential drugs targeting EMT-related signals (Table 3).

**Table 3:** Potential drugs targeting EMT-related modulators in clinical trials.

**Drug**	**Mechanism of action**	**Condition or disease**	**Phase**	**References**
Denosumab	Receptor activator of NF-κB ligands (RANKL)	Tenosynovial giant cell tumor	Launched in 2013	(45)
TK-006	Anti-TNFSF11 (RANKL)	Breast cancer-related bone metastases	I	(46)
WO-1066	STAT3 inhibitors, anti-PD-L1, Janus kinase (JAK) inhibitors	Melanoma, brain cancer	I	(47, 48)
DSP-0337	STAT3 inhibitor	Solid tumors	I	(49)
Danvatirsen	STAT3 expression inhibitors	Solid tumors	II	(50, 51)
OPB-111077	STAT3 ligands	Solid tumors	II	(52)
Napabucasin	STAT3 inhibitors	Colorectal carcinoma, pancreatic cancer	III	(53, 54)
PEGPH20	HIF1α inhibitors	Metastatic breast cancer	I/II	(55, 56)
CRLX-101	HIF1α inhibitors, DNA Topoisomerase I inhibitors	peritoneum cancer	II	(57, 58)

Modulators of transcription factors, such as nuclear factor-kappa B (NF-κB) and signaling transducer and activator of transcription 3 (STAT3) have made progress in clinical trials (59, 60). **Denosumab**, which is a macromolecule of humanized mAbs to receptor activator of NF-κB ligand (RANKL), was originally approved to treat and prevent postmenopausal osteoporosis in 2010 (45). **Denosumab** prevents RANKL binding to RANK, and blocks the development of osteoclasts, leading to restraining the resorption of bone. So far, phase III clinical studies have been ongoing for evaluating its therapeutic effect on metastatic non-small cell lung cancer (NSCLC) together with other chemotherapeutics. **TK-006** is another anti-RANKL antibody in early clinical development for the treatment of patients with bone metastases caused by breast cancer through hypodermic injection (46). In addition, **WO-1066** is a JAK/STAT3 (the Janus kinase/signal transducer and activator of tran-ions 3) signaling pathway and programmed cell death-ligand 1 (PD-L1) inhibitor, which is derived from the JAK2 inhibitor AG490. In 2019, the compound was granted an orphan drug designation in the U.S. for treating glioblastoma. Currently, the candidate is in phase I clinical trials for patients with melanoma or glioblastoma multiforme with brain metastases (47, 48). **DSP-0337**, **Danvatirsen** and **OPB-111077**, all inhibit STAT3 and are in phase I or II clinical trials to assess their therapeutic efficacy in solid tumors (49–52).

Hypoxia-inducible factor 1α (HIF1α) and β-catenin also regulate the expression of other transcription factors related to EMT (61, 62). **PEGPH20** (PEGylated recombinant human hyaluronidase PH20), which enzymatically degrades hyaluronic acid (HA), is currently being evaluated in phase II and III trials. It shows promising efficacy in preclinical and early clinical studies in the treatment of metastatic pancreatic carcinoma and other malignant tumors (55, 56). **CRLX-101** was proved to be a potent topoisomerase 1 and HIF1α inhibitor, which is a nanoparticle composed of CPT conjugated to a biocompatible copolymer of cyclodextrin and polyethylene glycol (PEG). Currently, CRLX101 is being evaluated in phase II clinical trials for several tumor types (58).

## Therapeutic Targeting of TME

Studies have shown that epigenetic changes of tumor cells caused by TME play a prominent role in tumor progression and invasion (1, 63). Tumor cells usually adapt to the changing external environment through changing the plasticity of tumor cells to meet the demand of tumor development. The research of relationship between TME and tumor plasticity is making progress in recent years (64). TME is composed of a complex mixture of ECM and various cells including cancer associated fibroblasts (CAFs) (65), cancer associated macrophages (CAMs) (66) and endothelial progenitor cells (EPCs) (67). Many components in ECM contribute to tumor growth. TME has become one of the key targets in tumor treatment due to its special pathophysiological characteristics and physicochemical properties (Table 4).

**Table 4:** Potential drugs targeting TME in clinical trials.

**Drug**	**Mechanism of action**	**Condition or disease**	**Phase**	**References**
Avadomide hydrochloride	TNF-α production inhibitor and cereblon inhibitors	Solid tumors	I/II	(68)
NIS-793	Anti-TGF-β	Solid tumors	I	(69)
AVID-200	TGF-β inhibitors	Solid tumors	I	(70)
SAR-439459	Anti-TGF-β	Solid tumors	I	(71)
Fresolimumab	Anti-TGF-β	Lung cancer	I/II	(72)
Simotinib hydrochloride	EGFR inhibitors	Lung cancer	I	(73)
Amcasertib	PDGFRα inhibitors	Hepatocellular carcinoma, cholangiocarcinoma	II	(74)
Olaratumab	Anti-CD140a (PDGFRα)	Soft tissue sarcoma	Launched in 2016	(75)
Cerdulatinib	JAK and Syk kinase inhibitors	Hematologic cancers	II	(76)
AZD-8055	mTORC1/2 inhibitors	Solid tumors	I	(77)
BI-860585	mTORC1/2 inhibitors	Solid tumors	I	(78)
DCBCI-0901	mTORC1/2 inhibitors Phosphatidylinositol 3-Kinase alpha (PI3Kα) inhibitors	Solid tumors	I	(79)
LXI-15029	mTORC1/2 inhibitors	Solid tumors	I	(80)
ABI-009	mTOR inhibitors	Metastatic cancer	II	(81)
Sapanisertib	mTORC1/2 inhibitors	Endometrial cancer	II	(82)
GSK-690693	Akt kinases 1 inhibitors	Lymphoma, solid tumors	I	(83)
ARQ-751	pan-Akt inhibitors	Solid tumors	I	(84)
TAS-117	PKB/Akt inhibitors	Solid tumors	II	(85)
Ipatasertib	PKB/Akt inhibitors	Prostate cancer	III	(86)
Siltuximab	Anti-IL6	Multiple myeloma	II	(87)
Sintilimab	Anti-PD-1	Lymphoma, Hodgkin's	Launched in 2019	(88)
Avelumab	Anti-PD-L1	Bladder and kidney cancer	Launched in 2017	(89)

Tumor necrosis factor alpha (TNF-α) could promote tumor growth via a PKCa- and AP-1-dependent pathway (90). **Avadomide** (**CC-122**) is a small molecule drug that inhibits both TNF-α and cereblon E3 ligase. The first-in-human phase I study, which evaluated the safety and clinical therapeutic effect of **avadomide** in patients with advanced solid tumors and others, showed acceptable safety and favorable pharmacokinetics (68). A**vadomide** is currently being evaluated in advanced melanoma in combination with Nivolumab. Transforming growth factor-β (TGF-β) signaling pathway is related to EMT in cancer cells (91). Therapeutic agents modulating the expression of TGF-β that are monoclonal antibodies include: **NIS-793** (a humanized anti-TGF-β monoclonal antibody), **AVID-200** (a recombinant inhibitor of TGF-β1 and TGF-β3), **SAR-439459** (targeting transforming TGF-β) and **fresolimumab** (a pan-specific human anti-TGF-β monoclonal antibody). Among these therapeutic agents, **fresolimumab** is able to neutralize all human isoforms of transforming TGF-β and being evaluated in phase I/II trials (72, 92).

Epidermal growth factor receptor (EGFR) regulates ECM and promotes cancer invasion (93). A small EGFR inhibitor **Simotinib** is used in phase I study to treat NSCLC (73). Platelet derived growth factor receptor alpha (PDGFRα), which contributes to fibroblast reprograming toward CAFs, plays a significant role in colorectal carcinogenesis (94). **Amcasertib**, a PDGFRα inhibitor and cancer stemness kinase inhibitor, is used to treat hepatocellular carcinoma and cholangiocarcinoma in phase II trials (74). Different from **Amcasertib**, **Lartruvo**(**R**) (**olaratumab**) is a fully humanized monoclonal antibody to neutralize PDGFRα. It was first launched in the U.S. for front-line treatment with doxorubicin in adults with soft tissue sarcoma in 2016 (75).

Some signaling pathways are also critical in cancer development. Janus kinase 1 (JAK1)/Rho kinase1 (ROCK1) signaling could promote fibroblast-dependent carcinoma cell invasion (95). **Cerdulatinib** is a small-molecule anti-cancer drug targeting JAK and syk kinase for the treatment of hematologic cancers (76). Liver kinase B1 (LKB1)/mammalian target of rapamycin (mTOR) signaling axis regulates ECM stiffness and participates in lung adenocarcinoma progression (96). Potential drugs such as **AZD-8055**, **BI-860585**, **DCBCI-0901**, **LXI-15029**, and **ABI-009** are in early clinical stage for various cancers (77–81). **Sapanisertib** is an orally and highly selective ATP-competitive inhibitor of mTORC1/2 and demonstrates satisfactory anticancer activity. The phase II study of **sapanisertib** in metastatic castration resistant prostate cancer was not entirely satisfactory likely because of dose reductions secondary to toxicity (82). In addition, abnormal expression of protein kinase B (PKB/Akt) is related to many cancers (97). **GSK-690693** (83), **ARQ-751** (84), and **TAS-117** (85) that can effectively treat solid tumors through inhibiting PKB/Akt are being evaluated in phase I and II clinical studies. **Ipatasertib** has been combined with other antitumor drugs to treat prostate cancer and breast cancer and is undergoing an investigation in a phase III clinical trial (86).

With the exception of targets above, interleukin-6 (IL-6) showed high expression in prostate cancer (98). **Siltuximab**, a chimeric monoclonal antibody, was first launched in 2014 to treat HIV-negative and Human Herpes Virus-8 negative multicentric Castleman's disease. Its tight binding to IL-6 inhibits IL-6 bioactivity and thus causes apoptosis of tumor cell. Recently, a phase II clinical trial of **siltuximab** was conducted for the treatment of multiple myeloma (87). Others like immunity-related programmed cell death receptor-1 (PD-1) and PD-L1 inhibitors show satisfied antitumor effects by restoring antitumor immunity. **Sintilimab** is a fully human IgG4 mAb, which blocks the interaction of PD-1 with PD-L1 and PL-L2 (88). It was firstly approved in China to treat classical Hodgkin's lymphoma. **Avelumab**, an anti-PD-L1 antibody, was approved by the FDA in 2019 for first-line treatment of advanced renal cell carcinoma together with axitinib (89).

## VM Related Targets and Therapeutic Agents

VM refers to a tumor microcirculation pattern that tumor cells aggregate, migrate and remodel to form a vascular-like structure based on the adhesion of ECM. VM differs from traditional endothelial tumor angiogenesis and plays a crucial role in tumor invasion and spreading. It is worth noting that there is an obvious increase of EMT-related regulators and transcription factors in VM, which indicates the crucial rule of EMT in VM formation (99). VM has been observed in a broad range of tumor types such as prostate cancer, malignant glioma, and melanoma (100). Currently, certain mechanism of VM formation remains matters of frenetic investigation and the mechanism of VM formation mainly include TME, EMT, tumor plasticity, RNA, and other regulators (100). Because VM is important for tumor progression, targeted therapies related to VM could also be a promising antitumor strategy to reducing tumor plasticity.

The major signaling molecules participating in VM formation and promising drugs are summarized in Table 5. Histone deacetylases inhibitor (HDACi) inhibits key molecule MMP-2 in PI3K-MMPs-Ln-5γ2 signaling pathway to block VM formation (112). **Panobinostat lactate**, which is lunched in 2015, is a first-line HDAC inhibitor applied in combination with bortezomib and dexamethasone to the treatment of multiple myeloma (113). **Panobinostat lactate** is not only a HDAC inhibitor but also a pan-deacetylase inhibitor. The pharmacokinetics of **panobinostat lactate** is affected by some factors such as hepatic impairment. HDAC inhibitor **romidepsin**, which is launched in 2010, could cause cell cycle arrest, differentiation and apoptosis in various cancer cells and is used for the treatment of cutaneous T-cell lymphoma (103). **OKI-179** and **remetinostat** are HDAC inhibitors in early clinical development (101, 102).

**Table 5:** Potential drugs targeting VM in clinical trials.

**Drug**	**Mechanism of action**	**Condition or disease**	**Phase**	**References**
OKI-179	HDAC inhibitors	Solid tumor	I	(101)
Remetinostat	HDAC inhibitors	Cutaneous T-cell lymphoma	II	(102)
Romidepsin	HDAC inhibitors	Cutaneous T-cell lymphoma, peripheral T-cell lymphoma	Launched in 2010	(103)
Panobinostat lactate	HDAC inhibitors	Multiple myeloma	Launched in 2015	(104)
MEN-1611	PI3K inhibitors	Breast cancer	I	(105)
HMPL-689	PI3Kδ inhibitors	B-cell lymphoma	I	(106)
Gedatolisib	PI3K/mTOR inhibitors	Acute myeloid leukemia, solid tumors	II	(107)
GDC-0980	PI3K/mTOR inhibitors	Prostate cancer	II	(108)
Buparlisib	PI3K inhibitors	HNSCC	III	(109)
Copanlisib hydrochloride	PI3K inhibitors	Lymphoma	Launched in 2017	(110)
siRNA-EphA2-DOPC	EphA2 inhibitors	Solid tumors	I	(111)

Phosphatidylinositide 3-kinases (PI3K) participate in VM formation by activating matrix metalloproteinases (MMPs) (114). The PI3Kα/δ inhibitor **copanlisib hydrochloride** was launched in 2017 as a treatment for relapsed follicular lymphoma in patients receiving two or more prior therapy regimens (110). **Copanlisib** characterizes low risk of PK-related pharmacological interaction due to reduced oxidation metabolism and unchanged excretion of copanlisib. Other PI3K inhibitors in clinical trials include **MEN-1611** (phase I for breast cancer), **HMPL-689** (phase I for B-cell lymphoma), **Gedatolisib** (phase II for acute myeloid leukemia and solid tumors), **GDC-0980** (phase II for prostate cancer) and **Buparlisib** (phase III in patients with head and neck squamous cell carcinoma, HNSCC) (105–109).

VE-cadherin mediates the activities of epithelial cell kinase (Eck/EphA2) to affect the formation of VM (115). EphA2 interacts with cell membrane surface ligands by phosphorylation and regulates the extracellular expression of protein kinases ERK and focal adhesion kinase FAK to activate PI3K (116, 117). **SiRNA-EphA2-DOPC** is a small interfering RNA targeting EphA2 loaded in neutral 1,2-dioleoyl-sn-glycero-3-phosphocholin (DOPC) liposomes (111). **SiRNA-EphA2-DOPC** reaches to tumor site by interacting with endothelial cells of tumor vasculature. As an EphA2 inhibitor, **siRNA-EphA2-DOPC** is in early clinical investigations to treat recurrent and advanced solid tumors.

## Therapeutic Targeting of Neoplasm Metabolic Pathways

Cancer cells reprogram metabolic pathways by oncogenic mutations, result in enhanced demand of nutrient uptake to supply anabolic metabolism. Not only must energy production and consumption processes in cancer cells be balanced to sustain tumor growth, but also cancer cells have to adapt to the changes in nutrition and oxygen supply caused by their rapid growth. Hence, malignant cells exhibit metabolic flexibility for them to exist and develop. Different from normal cells, cancer cells are more dependent on anaerobic glycolysis even in a sufficient oxygen supply environment, called Warburg effect (118). HIF-1α is crucial for anaerobic glycolysis under oxygen free conditions. Tumor suppressor liver kinase B1 (LKB1) regulates HIF-1α-dependent metabolic reprogramming (119). Recent studies have shown that Pyruvate kinase M2 (PKM2) plays a crucial part in the plasticity of cancer metabolism, and up regulation of PKM2 leads to oxidative metabolism (120). **Dimethylaminomicheliolide** (DMAMCL), a PKM2 activator, is a prodrug of micheliolide (MCL) that suppresses tumor growth and targets CSCs in the form of guaianolide sesquiterpene lactone. **Dimethylaminomicheliolide** could inhibit inflammation and tumor growth by releasing MCL into plasma. Early clinical trial using **Dimethylaminomicheliolide** for patients with solid tumors is being conducted (Table 6) (121).

**Table 6:** Potential drugs targeting neoplasm metabolic pathways in clinical trials.

**Drug**	**Mechanism of action**	**Condition or disease**	**Phase**	**References**
Dimethylamino-!!breakmicheliolide	PKM2 activators	Solid tumors	I	(121)
Acadesine	AMPK activators	Multiple myeloma therapy	I/II	(122)

In addition to this, oxidative phosphorylation plays an important role in cancer metabolism. Oxidative phosphorylation is mainly regulated by AMP-activated protein kinase (AMPK) (123). As an AMPK activator, **acadesine** increases the availability of adenosine in tissues under ischemic conditions and shows antitumor activity. **Acadesine** causes B cells apoptosis selectively in chronic lymphocytic leukemia (CLL) and phase I/II studies are being tested for sieving out the best methods for the treatment of resistant/refractory B-cell chronic lymphocytic leukemia (122). Metabolic plasticity of cancer triggers the adaptive “metabolic switch” needed for cancer development. Mechanism of metabolic switch provides insights into therapies, which could be used to target cancer development.

## Conclusions

Tumor plasticity provides new explanation for the mechanisms of drug resistance, metastasis and recurrence of neoplasm. Interfering tumor plasticity is becoming strategies to treat malignant tumors. The drugs in clinical trials that targeting tumor plasticity are still on intense research. However, targeted therapy also has some limitations that most drugs could only be effective on a small part of tumors of genetic transformation and engender drug resistance after a period of time of taking drugs. How to find effective multi-targeted inhibitors or combine with traditional chemotherapeutic drugs and other therapeutics like photodynamic or photothermal therapy become particularly important. The quest for new therapeutic targets toward tumor plasticity continues to be a great impetus to promote cancer treatment.

## Author Contributions

DW, X-GY, and L-CZ wrote the draft. Y-JW and Y-YL edited the manuscript. All authors read and approved the final version of manuscript.

### Conflict of Interest Statement

The authors declare that the research was conducted in the absence of any commercial or financial relationships that could be construed as a potential conflict of interest.
